# Evaluation of Projection Images for Visual Quality Control of Automated Left and Right Lung Segmentations on T1-Weighted MRI in Large-Scale Clinical Cohort Studies

**DOI:** 10.3390/tomography11120135

**Published:** 2025-11-29

**Authors:** Tobias Norajitra, Christopher L. Schlett, Ricarda von Krüchten, Prerana Agarwal, Ashis Ravindran, Thuy Duong Do, Lisa Kausch, Stefan Karrasch, Hans-Ulrich Kauczor, Klaus Maier-Hein, Claudius Melzig

**Affiliations:** 1 Division of Medical Image Computing, German Cancer Research Center (DKFZ), Im Neuenheimer Feld 280, 69120 Heidelberg, Germany; 2 Translational Lung Research Center Heidelberg (TLRC), Member of the German Center for Lung Research (DZL), University of Heidelberg, Im Neuenheimer Feld 420, 69120 Heidelberg, Germany; 3 Department of Diagnostic and Interventional Radiology, University Medical Center Freiburg, Faculty of Medicine, University of Freiburg, 79106 Freiburg, Germany; 4 Clinic for Diagnostic and Interventional Radiology, Heidelberg University Hospital, Im Neuenheimer Feld 420, 69120 Heidelberg, Germany; 5 Department of Nuclear Medicine, Heidelberg University Hospital, Im Neuenheimer Feld 400, 69120 Heidelberg, Germany; 6 Institute and Clinic for Occupational, Social and Environmental Medicine, LMU University Hospital, LMU Munich, 81377 Munich, Germany; 7 Institute of Epidemiology, Helmholtz Munich—German Research Center for Environmental Health, 85764 Neuherberg, Germany; 8 Comprehensive Pneumology Center Munich (CPC-M), Member of the German Center for Lung Research (DZL), 81377 Munich, Germany

**Keywords:** magnetic resonance imaging, cohort study, lung, deep learning, image processing

## Abstract

**Background/Objectives**: To assess diagnostic accuracy of two-dimensional (2D) projection methods for rapid visual quality control of automated volumetric (3D) lung segmentations compared with slice-based 3D review of segmentation results for application in large-scale studies. **Methods**: Segmentation of right and left lungs on T1-weighted MRI of 300 participants of the German National Cohort (NAKO) study was performed using the nnU-NET framework. Three variants of 2D projection images of segmentation masks were created: maximum intensity projection (MIP) using pseudo-chromadepth encoding with different color spectra for right and left lung (Colored_MIP) and standard deviation projection of segmentation mask outlines, encoded in black-and-white (Gray_outline) or using color-encoding (Colored_outline). The worst of two ratings by two independent raters conducting slice-based review for segmentation errors on underlying imaging data and review for mislabeling errors served as the standard of reference. All variants were evaluated by five raters each for identification of segmentation errors and the majority rating was used as index test. The time required for review was recorded and diagnostic accuracies were calculated. **Results**: Sensitivities of Colored_MIP, Colored_outline and Gray_outline were 88.2% [95%-CI 78.7%; 94.4%], 89.5% [80.3%; 95.3%] and 78.9% [68.1%; 87.5%]; specificities were 98.7% [96.1%; 99.7%], 96.4% [93.1%; 98.5%] and 98.7% [96.1%; 99.7%]; and F1-scores were 0.918, 0.895 and 0.863, respectively. Mean time per case and rater required for evaluation was 2.8 ± 0.9 s for Colored_outline, 1.7 ± 0.1 s for Colored_MIP, and 2.0 ± 0.4 s for Gray_outline. **Conclusions**: The 2D segmentation mask projection images enabled the detection of segmentation errors of automated 3D segmentations of left and right lungs based on MRI with high diagnostic accuracy, especially when using color-encoding. The method enabled evaluation within a matter of seconds per case. Segmentation mask projection images may assist in visual quality control of automated segmentations in large-scale studies.

## Simple Summary

Deep learning-based image segmentation is increasingly being used for research and clinical applications. However, quality control of segmentation results remains a challenge, particularly in specialized large-scale applications such as cohort-studies. We evaluated a visual approach to quality control of automated MRI-based left and right lung segmentations using coronal and axial projection images. The method enabled quality control within a matter of seconds per case and resulted in high diagnostic accuracy compared with slice-based three-dimensional review of segmentations. The method could prove valuable for future clinical and research applications and warrants further investigation.

## 1. Introduction

Automated deep learning-based volumetric (3D) medical image segmentation based on a small number of expert-annotated ground truth datasets is increasingly being used and has enabled semantic segmentation in large datasets, which would otherwise have required extensive, time-consuming manual labor [1,2]. However, validating the resulting automated segmentations, especially in large-scale datasets such as cohort studies, remains challenging. Large publicly available annotated datasets exist for certain tasks, such as CT-based lung segmentation, and can aid in external validation [3,4,5]. But these datasets are frequently not available for specialized segmentation tasks, imaging modalities such as certain MRI sequences or pathologies of interest. On the other hand, MRI is now increasingly being used in large population-based cohort studies around the world [6,7,8,9]. Semantic segmentation plays a key role in answering imaging-based research questions in these cases. However, visual quality control of every automated segmentation may not be feasible in large-scale cohort studies, and quality control of select cases cannot rule out the falsification of subsequent analyses by erroneous segmentations. Only few studies reported on the use of visual quality control in large cohort studies [2,10,11]. Various algorithmic approaches have been published in recent years to address this issue, for example, by quantifying uncertainty within a segmentation algorithm or an ensemble of segmentation algorithms [12,13]. While these may help identify potentially erroneous segmentations and provide an indication of overall segmentation performance, significant segmentation errors may still be missed. Visual quality control on a case-by-case basis, therefore, remains desirable. Few previous publications presented and evaluated dedicated software tools that streamline the process of loading imaging data and associated segmentation masks for manual slice-based 3D quality control [2,14,15]. However, evaluation of numerous slices per case still accumulates to a significant amount of time in large-scale studies like the German National Cohort (NAKO) with ~30,000 participants undergoing MRI scans or the UK Biobank imaging study which includes imaging of ~100,000 participants [6,7]. Two-dimensional projection images for visualization and analysis of three-dimensional imaging data, especially maximum intensity projections, are routinely used for visualization and aid assessment of computed tomography and MRI examinations, especially angiographic examinations [16,17]. Standard deviation projection images have been previously reported to improve visualization and outlining of cells on microscopy images [18]. To our knowledge, only one prior study reported on the use of projection images for visual quality control of automated segmentations; however, the accuracy of the method compared to slice-based review was not reported [2]. We, therefore, assessed the diagnostic accuracy of different 2D projection images of 3D right and left lung segmentation masks based on MRI examinations of a large-scale cohort study for rapid identification of segmentation errors compared with slice-based review.

## 2. Materials and Methods

### 2.1. Patients

The lung segmentation algorithm was trained on MRI data of two national multicenter studies: the imaging-based sub-study of the COSYCONET cohort study on chronic obstructive pulmonary disease (“COPD and SYstemic consequences-COmorbidities NETwork,” NCT01245933; “Image-based structural and functional phenotyping of the COSYCONET cohort using MRI and CT (MR-COPD),” NCT02629432) and the population-based NAKO [19,20,21]. The present analysis was solely performed on lung segmentations of NAKO. NAKO is an ongoing population-based study within a network of 25 institutions at 18 regional examination sites. The main objective is the investigation of risk factors for chronic diseases. The baseline assessment enrolled 205,415 participants from the general population (age 19–74 years) between 2014 and 2019, of which 30,861 also participated in the NAKO MRI study. This imaging sub-study was carried out at five imaging centers. For the present analysis, automated lung segmentation was performed on MRI data of 11,190 participants available at the time of the investigation, enrolled until 31 December 2016. Segmentation visualization, as assessed in the present manuscript, was evaluated in 300 of these participants, which were selected based on model uncertainty from the neural network ensemble used for automatic segmentation. Uncertainty was quantified based on the volumetric disagreement among model outputs: $U\left(x,y\right)\;=\;1\;-\;\frac{1}{k}\sum_{i\neq j}Dice\left(i,j\right)$ for binary segmentation *y* on image *x*, Dice overlap of pairwise segmentation masks *Dice(i, j)* generated across all ensemble models, and the number of mask combinations *k*. Three subsets of 100 cases were then selected, representing the highest, lowest and median uncertainty measurements. The selection was aimed at representing (1) cases with a high prevalence of salient errors, (2) cases with a low prevalence of subtle errors, and (3) the most frequent cases within the underlying dataset. The selection was not intended to represent the dataset as a whole. Written informed consent was obtained from all study participants. The present analysis was approved by the NAKO Use and Access Committee and the steering committee of the COSYCONET study. Ethical approval was obtained from the Ethics Committee of the Medical Faculty of the University of XXXX (S-193/2021).

### 2.2. MR Imaging

**Figure 1:**
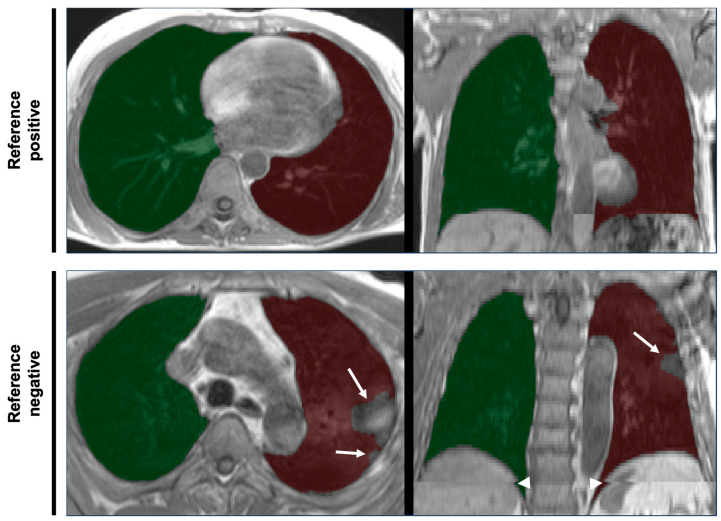
Examples of thoracic MRI data and overlying lung segmentation masks. Axial slices (left column) and coronal views (right column) of the thoracic part of axially acquired T1-weighted 3D VIBE two-point DIXON images of thorax and abdomen of two different participants of the NAKO study. Translucent overlays of right (green) and left (red) lung segmentation masks as results of deep learning-based automated segmentation are shown. The quality of the lung segmentation of the participant in the top row was deemed sufficient for further analysis (reference positive). The bottom row shows an example of erroneous segmentation according to the reference standard (reference negative). Note the exclusion of consolidated parts of the lungs (arrows) and the spatial inconsistencies (composing artifacts, arrowheads) resulting from the stitching of separate acqusitions for the lower and upper part of the scan volume.

Whole-body MRI scans as part of NAKO were conducted at five study centers and are described in detail elsewhere [6]. In brief, lungs were covered in an axially acquired T1-weighted 3D VIBE two-point DIXON sequence of thorax and abdomen in inspiratory breath-hold (slice thickness 3.0 mm, voxel size in-plane 1.4 × 1.4 mm) using 3T MRI scanners (MAGNETOM Skyra, Siemens Healthineers AG, Forchheim, Germany). Examples are provided in Figure 1.

### 2.3. Automated Lung Segmentation

Automated, deep learning-based lung segmentation was performed using the nnU-NET framework in 3D full resolution mode [22]. Training of segmentation models was based on manual ground truth segmentations of stratified samples from NAKO (*n* = 16) and COSYCONET (*n* = 13) created by two medical experts. Right and left lungs were segmented separately and the lung hili excluded. The cases used for training of nnU-NET were not included in the present analysis of segmentation mask projection images. The training set size was determined heuristically, guided by the observed difficulty of the lung boundary delineation task. The objective was to establish reliable segmentation performance for the majority of the dataset, while allowing effective sampling of error cases for the presented quality control. Preprocessing was performed by nnU-Net. Specifically, images were cropped to regions containing non-zero intensities, resampled to a voxel spacing of 1.4 × 1.4 × 3.0 mm, and subjected to z-score normalization. Data augmentation was applied, comprising random rotation, scaling, elastic deformation, and gamma correction during training, and mirroring during training and application. No post-processing was performed. The U-Net architecture was configured using fundamental building blocks of 3 × 3 convolution, instance normalization and Leaky ReLU activations, with the number of feature channels ranging from 32 to 320. Training was performed using stochastic gradient descent with Nesterov momentum (0.99) and an initial learning rate of 0.01. The sum of cross-entropy and Dice loss was used as loss function. An ensemble of five networks was trained on disjoint subsets created through 5-fold cross-validation of the annotated images and subsequently used for inference. Train and test datasets were strictly separated on the subject level using unique subject identifiers.

### 2.4. Segmentation Mask Projection Images

Three different variants of projection images were created (Colored_MIP, Colored_outline, and Gray_outline), using either maximum intensity projection (MIP) of segmentation masks (Colored_MIP) or standard deviation projection of the isosurface between foreground and background voxels of the binary segmentation masks (Colored_outline, Gray_outline) of right and left lungs in axial and coronal orientation. For Colored_MIP and Colored_outline, a pseudo-chromadepth approach was chosen to improve depth perception [23,24,25]. In order to also color-encode the labeling of right and left lung, two different color spectra for the right (mpl-viridis) and the left lung (mpl-plasma) were used in these cases. For projection, only slices containing lung voxels were selected in coronal and axial orientation. Resulting slice numbers were then linearly mapped to the respective color spectra. For comparison with grayscale projection images (Gray_outline), segmentation surface voxels were encoded in white and all other voxels in black. For subsequent z-stack projections using maximum intensity projection, each pixel of the resulting 2D image represents the maximum intensity of all voxels in the same x,y-position along the z-stack. In case of color-encoding, this refers to the maximum intensity per RGB-color channel. For standard deviation projections, the pixel values were calculated to represent the standard deviation of voxel intensities over the z-stack in the same position according to the formula $\sigma \;=\;\sqrt{\frac{1}{N-1}\sum_{i=1}^{N}{\left(I_{i}-\mu \right)}^{2}}$, where *I_i_* is the pixel intensity in slice *i* of the current color channel of the z-stack, *μ* is the mean of the pixel intensities along the z-stack, and *N* is the number of slices in the z-stack. Axial and coronal projections were composed side by side to create a single panel for every segmentation mask. Projection images were created using FIJI (version 2.1.0/1.53c) and the Z-stack Depth Color Code plugin (version 0.0.2) [26,27]. The corresponding macro script used to generate the projection images is provided as Supplemental Material.

### 2.5. Visual Segmentation Quality Assessment

Five raters (C.M., R.v.K., C.S., T.N., F.A.), of which four were radiologists and one (T.N.) a computer scientist with each at least 5 years of experience in lung imaging and segmentation, independently evaluated lung segmentation mask projections. Images were reviewed in random order in rapid succession using an in-house-developed web browser-based software that displayed axial and coronal projection images side by side on a 50% gray background, one case at a time. Left and right arrow keys on the computer keyboard were then used to rate the segmentation as either successful or erroneous. The next projection image was presented immediately after the rating of the last and respective ratings were automatically recorded for later analysis. Raters always evaluated the 300 cases in one continuous session per projection method. The total time required for each complete reading session was recorded manually by each rater and divided by 300 to calculate the average read time per case. Colored_outline images were rated first. The reads were repeated by all raters after a wash-out period of 1 week to assess intra-rater reliability. Gray_outline and then Colored_MIP images were rated after another wash-out interval of at least 2 weeks. The raters were blinded to any additional information and specifically instructed to “identify cases with any significant error in lung segmentation that hinders downstream analyses in the large MRI cohort study. Errors may include: incorrect anatomical segmentation, segmentation error due to possibly inadequate image quality, segmentation of non-lung structures (such as stomach), or the (partial) interchange of right and left lung labels. Swipe left in case of significant segmentation error, otherwise swipe right.”.

### 2.6. Reference Standard

One rater (R.v.K.) with more than 5 years of experience in lung imaging and research reviewed original DICOM data and an overlay of the corresponding segmentation masks using dedicated software (NORA Medical Imaging Platform Project, University Medical Center Freiburg, Freiburg, Germany). Slice-based review of the 3D segmentation masks for segmentation errors was performed using axial, coronal and sagittal view planes, and according to the same criteria used for projection images: errors that would significantly impact further analyses. Additionally, a second rater (C.M.) reviewed segmentation masks specifically for mislabeling of right and left lung, and any mislabeling was considered a significant segmentation error. Both raters conducted the review in random order, independently and blinded for the results of the other. Identified segmentation errors were assigned to the following categories: over-/under-segmentation of lung boundaries, exclusion of lung pathology, inclusion of distant organs, off-target stitching and partial or complete left–right mislabeling of the lungs. The worst rating of both reads was used for each case as the standard of reference. To avoid recall bias, as both raters were also involved in the evaluation of the index test, the ratings for reference standard and index test were separated by at least 6 months.

### 2.7. Statistical Analysis

Normal distribution was assessed using QQ-plots and the Kolmogorov–Smirnov test. Group comparisons were performed using Student’s *t*-test or chi-squared test where appropriate. A receiver operating characteristic curve (ROC) was created for each projection method over the sum of ratings of the five individual raters and corresponding areas under the curve (AUC) and corresponding 95% confidence intervals calculated according to the method by DeLong. Measures of diagnostic accuracy (sensitivity, specificity, accuracy and F1-score) were calculated and confidence intervals calculated according to the Clopper–Pearson method. Sensitivities and specificities were compared using Cochran’s Q test for multiple classifiers followed by post hoc pairwise McNemar tests with Bonferroni adjustment of *p*-values for multiple comparisons. Inter-rater reliability was calculated using Fleiss’ Kappa, and Cohen’s Kappa was used to assess intra-rater reliability. Kappa measures were interpreted as follows [28]: <0.00 poor, 0.00–0.20 slight, 0.21–0.40 fair, 0.41–0.60 moderate, 0.61–0.80 substantial, 0.81–1.00 almost perfect. All statistical analyses were performed using R Version 4.0.2 (R Foundation for Statistical Computing, Vienna, Austria).

## 3. Results

### 3.1. Study Sample

Demographics of the study sample are summarized in Table 1. Segmentation errors were found in 76/300 (25.3%) cases. The types and frequencies of observed segmentation errors according to the reference standard are shown in Table 2. Participants with lung segmentation error according to the reference standard were slightly heavier (82.8 ± 28.2 kg vs. 75.0 ± 17.1 kg, *p* = 0.024). Mean lung volume of the study sample based on lung segmentations was 4139.1 ± 1096.2 mL (range 1630.4–8439.4 mL). Lung volumes did not differ significantly between patients with and without lung segmentation error (4045.9 ± 1119.0 mL vs. 4170.8 ± 1089.1 mL, *p* = 0.40).

### 3.2. Accuracy

Accuracies of the five individual raters for the detection of segmentation errors is summarized in Table 3.

**Figure 2:**
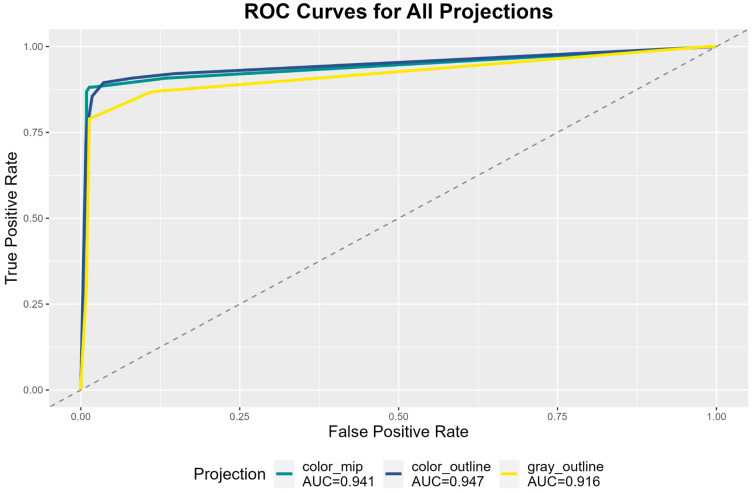
Receiver operating characteristic (ROC) curves and corresponding areas under the curve (AUC) for three different projection methods over the sum of ratings of five individual raters for the identification of segmentation errors.

An ROC analysis was conducted for each projection method over the corresponding sum of the binary ratings of the five independent raters (Figure 2). Resulting AUCs were 0.941 (95%-CI 0.904–0.979), 0.947 (95%-CI 0.911–0.982) and 0.916 (95%-CI 0.874–0.959) for Colored_MIP, Colored_outline and Gray_outline, respectively. The maximum Youden-index was reached at a threshold of >/≤ 2.5 positive ratings with Youden-indices of 186.8, 185.9 and 177.6 for Colored_MIP, Colored_outline and Gray_outline, respectively.

**Figure 3:**
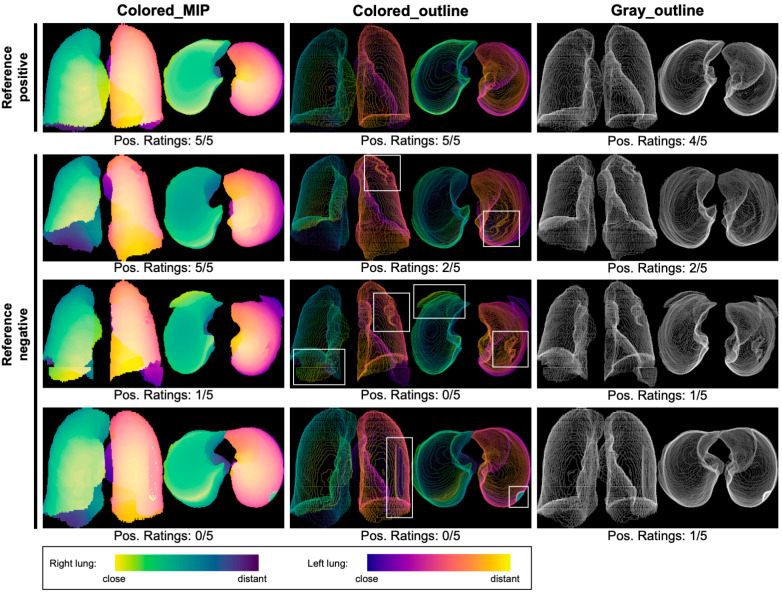
Examples of projection images and observed segmentation errors. Three segmentation mask projections were evaluated: solid segmentation mask projection using maximum intensity projection (MIP) and color-coding of left and right lung as well as depth along the respective *z*-axis (left column “Colored MIP”), standard deviation projection of the isosurface between foreground and background voxels of the binary segmentation masks using either color-coding of left and right lung as well as depth along the respective *z*-axis (middle column “Colored outline”) or binary encoding with lung voxels represented in white and all other voxels represented in black (right column “Gray outline”). The first and third row are based on the segmentation masks shown in Figure 1. The top row demonstrates a segmentation without significant errors according to the reference standard. The lower three rows show examples of segmentation errors, highlighted by bounding boxes in the middle column: over-/under-segmentation of parts of the lung (row two), exclusion of lung pathology (consolidation) and off-target stitching (third row) and right–left mislabeling of parts of the lung (bottom row).

Consequently, the majority rating of the five raters was used to assess accuracies of the three projection methods; see examples provided in Figure 3. Examples of a potential color-vision-deficiency-safe version using yellow and cyan palettes for right and left lung are provided in Figure S2. This version was, however, not evaluated in the present analysis. Accuracies of majority ratings are summarized in Table 4. Sensitivity was highest for the Colored_outline method (89.5%, 95% confidence interval (CI) 80.3–95.3%), followed by Colored_MIP (88.2%, CI 78.7–94.4%) and Gray_outline (78.9%, CI 68.1–87.5%). The differences in sensitivity between the three projection methods were statistically significant (*p* = 0.006) and pairwise post hoc tests revealed a statistically significant difference between Colored_outline and Gray_outline (*p* = 0.04).

The highest specificity was observed for Colored_MIP (98.7%, CI 96.1–99.7%) and Gray_outline images (98.7%, CI 96.1–99.7%). Differences in specificity between the three methods were significant (*p* = 0.03); however, pairwise post hoc tests were not significant (all *p* > 0.05).

Overall accuracy and F1-score were highest for Colored_MIP (96.0%, CI 93.1–97.9%; F1-score 0.918), followed by Colored_outline (94.7%, CI 91.5–96.9%; F1-score 0.895) and Gray_outline (93.7%, CI 90.3–96.1%; F1-score 0.863).

Accuracy of the majority rating was also analyzed separately for the three different strata of the algorithm uncertainty metric that the study sample was based on (Table 5). Off 100 cases included in each stratum, 63% in the stratum with maximum uncertainty had segmentation errors according to the reference standard, whereas error rates of 6% and 7% were found for the median and minimum uncertainty strata, respectively. Distribution of the uncertainty metric in the study sample is shown in Figure S1.

Finally, sensitivity of majority ratings for different error categories were calculated (Table 6). Sensitivity for left–right mislabeling was 100% for both color-coding methods and 88.5% for Gray_outline. Sensitivities for other error categories varied between 50 and 100% and minor differences between the projection methods were found only for the category over-/under-segmentation of lung boundaries with slightly lower sensitivity for Gray_outline (79.4%) compared with 82.4% and 85.3% for Colored_MIP and Colored_outline, respectively.

### 3.3. Inter- and Intra-Rater Reliability

Inter-rater reliabilities based on Fleiss’ Kappa were almost perfect for Colored_outline and Colored_MIP (Kappa 0.82 and 0.87) and poor for Gray_outline (Kappa −0.01).

Intra-rater reliability was assessed for Colored_outline images based on Cohen’s Kappa. The Kappa values of all five raters were almost perfect with values ranging between 0.82 and 0.95.

### 3.4. Evaluation Time

Mean time required per case and rater for the evaluation of segmentations was 1.7 ± 0.1 s (1.6–1.9 s) for Colored_MIP projections, 2.8 ± 0.9 s (range 2.0–4.0 s) for Colored_outline images, and 2.0 ± 0.4 s (1.7–2.4 s) for Gray_outline images. Differences in evaluation time between the three projection methods were not statistically significant (*p* = 0.15).

## 4. Discussion

We evaluated three 2D visualization techniques for visual quality control of deep learning-based 3D segmentations of the right and left lungs on whole-body MRI scans. Accuracies were highest for the methods using color-coding for right and left lung segmentation and depth along the *z*-axis with accuracies of 96.0% and 94.7% for Colored_MIP and Colored_outline images, respectively, based on five raters’ readings. Inter-rater reliability was almost perfect for both color-coding methods (0.87 and 0.82) and poor for Gray_outline (−0.01). Intra-rater reliability, assessed for the Colored_outline method, was also almost perfect for all 5 raters. The mean time required per case and rater varied between 1.7 s and 2.8 s.

Our observation, that color-coding resulted in higher accuracy compared to black-and-white coding for the detection of semantic segmentation errors when assessing a multilabel segmentation (left lung, right lung, background), was to be expected. This was further supported by our observation that 100% of cases with left–right mislabeling error were detected by the methods using color-coding, whereas Gray_outline only detected 88.5% of these cases. Of course, these errors can only be identified if the quality control method allows differentiation of the corresponding labels. However, we observed a large variability in the accuracy of Gray_outline images among the raters, with two raters performing notably worse. We hypothesize this may be related to a priori knowledge about the possibility of partially mislabeled right and left lung, leading to islands of left lung labels inside the right lung mask and vice versa. While the lack of color-coding prohibits direct identification of the assigned label, the segmentation mask outlines highlight these areas. Therefore, related to the predefined order in which the projection methods were evaluated, some raters possibly interpreted them as mislabeling. On the other hand, sensitivities for non-left–right mislabeling errors were still high in the majority of categories, especially for the second most frequent error type “over-/under-segmentation of lung boundaries”, with sensitivities between 79.4% and 85.3%.

Based on the sampling strategy used in our study, we found a much higher error rate of 63% in the sample stratum with the highest algorithm uncertainty and associated high sensitivities and specificities of the projection methods between 85.7–92.1% and 83.8–94.6%, respectively. In comparison, accuracy was lower in the strata with the majority of cases according to the uncertainty metric (median uncertainty) and those with the lowest uncertainty and correspondingly expectedly low error prevalence of 7%. However, sensitivities of 71.4% each and specificities of 100% and 98.9% for Colored_MIP and Colored_outline were still high in these scenarios and may still provide added benefit for identification of significant errors.

Few publications have previously reported on the use of visualization techniques for quality control of 3D biomedical image segmentation. Volume rendering has been used previously to improve the validation of automated semantic segmentation of confocal microscopy imaging data [29]. However, the diagnostic performance of the resulting visualizations was not assessed. A recent publication on abdominal organ segmentation on MRI in two of the largest cohort studies, NAKO and UK Biobank, employed a similar approach to visual quality control, also incorporating MIP images of color-coded organ segmentation masks and underlying imaging data in axial, coronal and sagittal orientation [2]. The images for quality control of 20.000 study participants were all reviewed by one rater. The authors did not report on the accuracy of the chosen approach compared to a slice-based review of the segmentations [2]. Algorithm-derived methods for automated quality control of deep learning-based segmentations are being intensively investigated [30,31,32]. These approaches depend on large training datasets and demonstrated high performance especially in cases of severe segmentation errors. But smaller segmentation errors may not be detected as effectively, which supports the notion of a multimodal approach to quality control [33,34]. Specifically, ensemble uncertainty-based metrics appear promising; however, this has so far been primarily demonstrated for non-segmentation approaches or with limited predictive value for the actual segmentation accuracy [35,36]. Some publications have reported on algorithmic approaches investigating anatomic plausibility of resulting segmentations to detect segmentation errors [37] and point to an approach for segmentation quality control independent of underlying raw data, similar to the visual approach chosen in our study [38,39].

A recent publication presenting a new software tool for streamlined 3D slice-based review of medical imaging segmentations reported a review time for brain tumor segmentations on MRI of 11 s per case for one experienced radiation oncologist [15]. While not directly comparable due to different organ and segmentation tasks, this indicates the potential time savings of the presented 2D projection methods. We used the majority rating of five raters to finally decide on the quality of the segmentation, which prolongs the overall time invested per case. However, manual 3D segmentation and review of automated segmentations also frequently involve more than one rater due to unavoidable inter-rater variability [40]. Nevertheless, inter-rater reliability and accuracy were high for our proposed method and the investigated scenario and we believe the number of raters and potential correction–re-segmentation–re-evaluation cycles should be adjusted according to the specific use case.

A few limitations of this study have to be considered. The present evaluation of 2D projection images is limited to MRI-based segmentations of one pairwise organ of relatively simple shape. Diagnostic accuracy of the method may of course vary based on the specific segmentation task, anatomical region, segmentation algorithm and underlying imaging data. Generalizability of our findings is therefore limited. However, we feel color-coded projection images may be of potential use for any multilabel semantic seg-mentation task. Similarly, the variety of segmentation errors encountered will likely vary. While the observed sensitivity for non-left–right mislabeling errors varied between 50 and 100%, it has to be taken into account that some error categories were represented by only very few cases in our sample. Finally, we did not include the underlying imaging data in the visualizations and based our visual quality control approach solely on the subjective anatomical plausibility of the segmentation masks. This decision was based on a few example cases that led the authors to the impression that inclusion of the underlying imaging data in the projection images may not improve the process of visual quality control due to the increased complexity of the resulting images. Therefore, we believe the presented approach should not be interpreted as a single solution to the overall segmentation quality control problem of large-scale studies. Instead, a multi-rater visual quality control should complement algorithmically derived measures of segmentation accuracy and potentially slice-based spot-checking of segmentation results in small subsamples.

## 5. Conclusions

In conclusion, 2D segmentation mask projection images allowed for rapid quality control of automated volumetric segmentations of left and right lungs based on T1-weighted MRI in an uncertainty metric-based stratified sample with moderate to high diagnostic accuracies compared with slice-based review as a standard of reference, especially when using color-coding. Accuracy varied with algorithm uncertainty and type of segmentation error. The method demonstrated high inter-rater reliabilities and enabled evaluation within a matter of seconds per case. Segmentation mask projection images may be potentially useful in large-scale studies and external validation of semantic segmentation algorithms in general. However, evaluation of the method in other segmentation scenarios and imaging modalities as well as larger datasets is desirable.

## Figures and Tables

**Table 1:** Characteristics of the study sample.

	All Participants	Lung Segmentation Error	No Lung Segmentation Error	*p*-Value
N	300	76 (25.3%)	224 (74.6%)	
Age [years]	49.8 ± 12.0	49.4 ± 12.5	49.9 ± 11.8	0.40
Female	145 (48.3%)	33 (43.4%)	112 (50.0%)	0.39
Height [m]	1.73 ± 0.09	1.74 ± 0.09	1.73 ± 0.09	0.40
Weight [kg]	76.9 ± 20.7	82.8 ± 28.2	75.0 ± 17.1	0.024
BSA [m^2^]	1.90 ± 0.26	1.96 ± 0.33	1.88 ± 0.22	0.05
Mean lung volume * [mL]	4139.1 ± 1096.2	4045.9 ± 1119.0	4170.8 ± 1089.1	0.40

Values are presented as mean ± SD or number of participants with percentage in parentheses. *p*-values were calculated using Student’s *t*-test for continuous variables and chi-squared test for categorical variables. *: Lung volume based on automated lung segmentations. BSA = body surface area.

**Table 2:** Type and frequency of segmentation errors according to the reference standard.

Error Type	*n*	Frequency
Left–right mislabeling	61/76	80.3%
Over-/Under-segmentation of lung boundary	34/76	44.7%
Exclusion of lung pathology	5/76	6.6%
Off-target stitching	4/76	5.3%
Inclusion of distant organs	3/76	3.9%
Failed separation of right and left lung	1/76	1.3%

Values represent number of cases with observed segmentation error type in relation to number of cases with any segmentation error and corresponding frequency in percent.

**Table 3:** Accuracy of individual raters for detection of segmentation errors.

	Rater 1	Rater 2	Rater 3	Rater 4	Rater 5
Colored_MIP					
Sensitivity	90.8%	88.2%	82.9%	85.5%	86.8%
Specificity	92.4%	98.7%	99.1%	96.9%	94.6%
Accuracy	92.0%	96.0%	95.0%	94.0%	92.7%
F1-score	0.852	0.918	0.894	0.878	0.857
Colored_outline					
Sensitivity	88.2%	90.8%	78.9%	82.9%	92.1%
Specificity	94.6%	92.9%	99.1%	93.8%	90.6%
Accuracy	93.0%	92.3%	94.0%	91.0%	91.0%
F1-score	0.865	0.857	0.870	0.824	0.838
Gray_outline					
Sensitivity	23.7%	78.9%	77.6%	38.2%	78.9%
Specificity	95.1%	99.1%	94.6%	3.1%	96.0%
Accuracy	77.0%	94.0%	90.3%	12.0%	91.7%
F1-score	0.343	0.870	0.803	0.180	0.828

Sensitivity, specificity and accuracy are presented as percentages. MIP: maximum intensity projection.

**Table 4:** Diagnostic accuracy of majority rating from five raters, employing different segmentation mask projection methods, for detecting segmentation errors.

	Colored_MIP	Colored_Outline	Gray_Outline
Sensitivity	88.2% [78.7%; 94.4%]	89.5% [80.3%; 95.3%]	78.9% [68.1%; 87.5%]
Specificity	98.7% [96.1%; 99.7%]	96.4% [93.1%; 98.5%]	98.7% [96.1%; 99.7%]
Accuracy	96.0% [93.1%; 97.9%]	94.7% [91.5%; 96.9%]	93.7% [90.3%; 96.1%]
PPV	95.7% [88.0%; 99.1%]	89.5% [80.3%; 95.3%]	95.2% [86.7%; 99.0%]
NPV	96.1% [92.7%; 98.2%]	96.4% [93.1%; 98.4%]	93.2% [89.3%; 96.1%]
F1-score	0.918	0.895	0.863

Sensitivity, specificity and accuracy are presented as percentages with 95% confidence intervals in square brackets. MIP: maximum intensity projection, NPV: negative predictive value, PPV: positive predictive value.

**Table 5:** Diagnostic accuracy of majority rating from five raters, employing different segmentation mask projection methods, for detecting segmentation errors, stratified by algorithm uncertainty score.

Projection Method	Uncertainty Score Stratum	Sensitivity	Specificity
Colored_MIP	Max	58/63 (92.1%)	35/37 (94.6%)
Colored_outline	Max	59/63 (93.7%)	31/37 (83.8%)
Gray_outline	Max	54/63 (85.7%)	34/37 (91.9%)
Colored_MIP	Median	4/6 (66.7%)	93/94 (98.9%)
Colored_outline	Median	4/6 (66.7%)	93/94 (98.9%)
Gray_outline	Median	3/6 (50.0%)	94/94 (100.0%)
Colored_MIP	Min	5/7 (71.4%)	93/93 (100.0%)
Colored_outline	Min	5/7 (71.4%)	92/93 (98.9%)
Gray_outline	Min	3/7 (42.9%)	93/93 (100.0%)

Sensitivity and specificity are presented as number of cases with segmentation errors identified in relation to all cases with segmentation error per stratum and percentages in parentheses. MIP: maximum intensity projection.

**Table 6:** Sensitivity of majority rating from five raters, employing different segmentation mask projection methods, for detection of specific segmentation error categories.

Error Category	Colored_MIP	Colored_Outline	Gray_Outline
Left–right mislabeling	61/61 (100%)	61/61 (100%)	54/61 (88.5%)
Over-/Under-segmentation of lung boundaries	28/34 (82.4%)	29/34 (85.3%)	27/34 (79.4%)
Exclusion of lung pathology	4/5 (80%)	4/5 (80%)	4/5 (80%)
Off-target stitching	2/4 (50%)	2/4 (50%)	2/4 (50%)
Inclusion of distant organs	3/3 (100%)	3/3 (100%)	3/3 (100%)
Failed separation of right and left lung	1/1 (100%)	1/1 (100%)	1/1 (100%)

Sensitivities are presented as number of cases with segmentation errors identified in relation to all cases with the respective segmentation error category and percentages in parentheses. MIP: maximum intensity projection.

## Data Availability

Restrictions apply to the availability of these data. The underlying imaging data of the NAKO study are available from the NAKO data transfer site https://transfer.nako.de (accessed on 5 October 2025) upon a written proposal, pending scientific review, and a signed cooperation agreement. Data from the German COPD cohort COSYCONET used in this study is available upon written proposal and approval by the steering committee, as detailed on the corresponding website www.asconet.net (accessed on 5 October 2025). The derived lung segmentations datasets and projection images analyzed in this article are not readily available because the data are part of an ongoing study, but they will be available via the NAKO transfer site committee upon application in the future.

## Supplementary Materials

The following supporting information can be downloaded at: https://www.mdpi.com/article/10.3390/tomography11120135/s1, Figure S1: Violin plot illustrating the distribution of the algorithm uncertainty metric in the study. Figure S2: Examples of projection images using a color-vision-deficiency-safe palette. Segmentation visualization.ijm: FIJI-macro for creation of projection images.

## Abbreviations

The following abbreviations are used in this manuscript:

2D	2-dimensional
3D	3-dimensional
MRI	Magnetic resonance imaging
MIP	Maximum intensity projection
