# Supplementary material for: Evaluation of Projection Images for Visual Quality Control of Automated Left and Right Lung Segmentations on T1-Weighted MRI in Large-Scale Clinical Cohort Studies
Source: Tomography. 2025 Nov 29;11(12):135. doi: 10.3390/tomography11120135 (PMC12736869; doi:10.3390/tomography11120135)
Supplement: Supplementary file 1 [file tomography-11-00135-s001.zip › Figure S1.pdf]

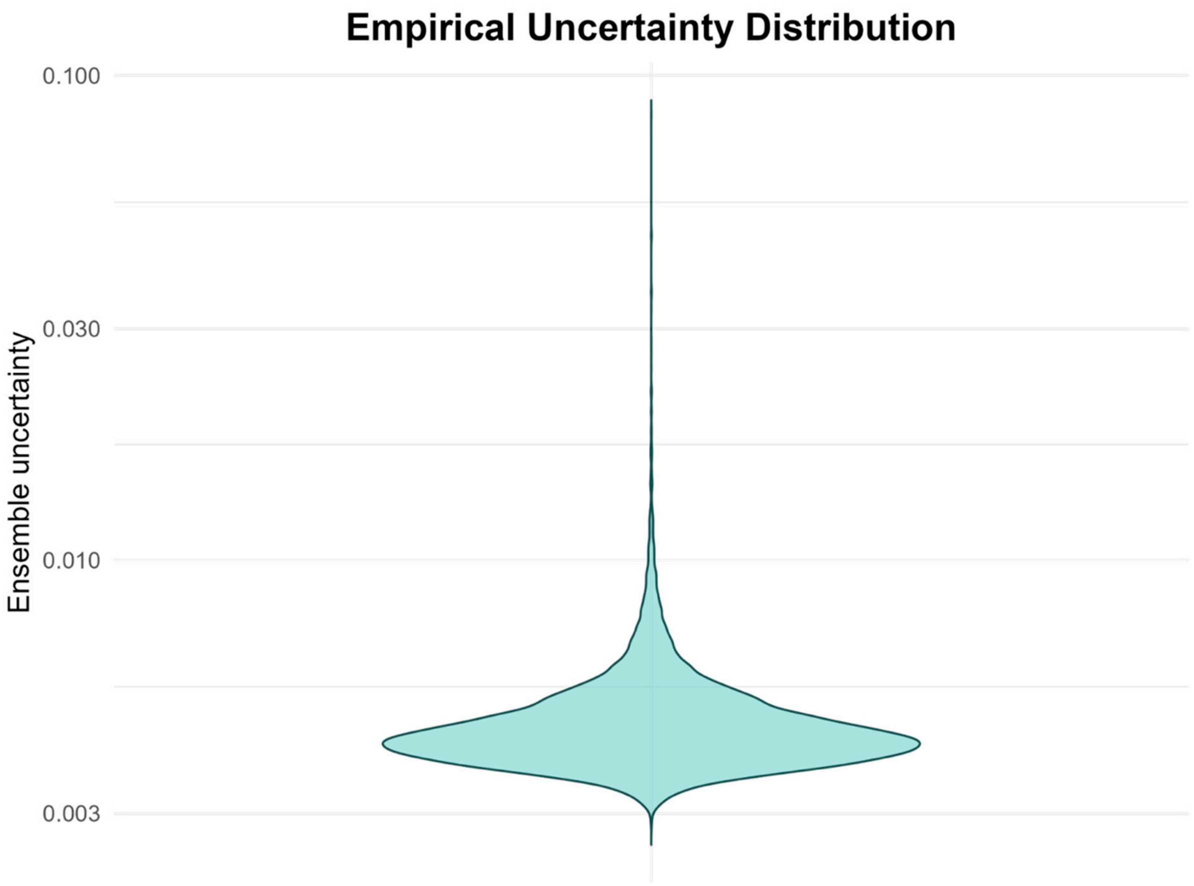

Figure S1. Violin plot illustrating the distribution of the algorithm uncertainty metric in the study.
