# Supplementary material for: Evaluation of Projection Images for Visual Quality Control of Automated Left and Right Lung Segmentations on T1-Weighted MRI in Large-Scale Clinical Cohort Studies
Source: Tomography. 2025 Nov 29;11(12):135. doi: 10.3390/tomography11120135 (PMC12736869; doi:10.3390/tomography11120135)
Supplement: Supplementary file 1 [file tomography-11-00135-s001.zip › Figure S2.pdf]

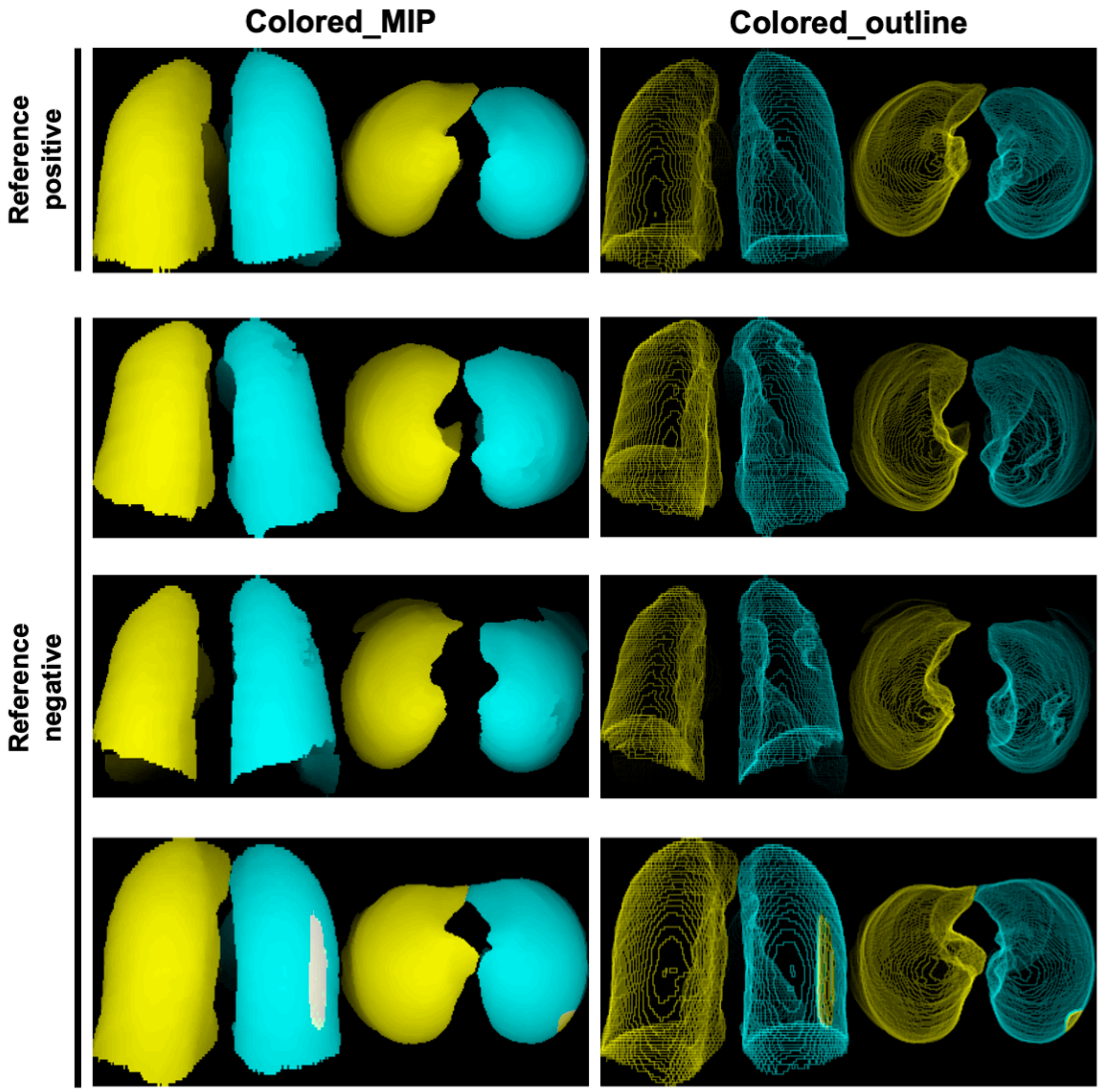

Figure S2. Examples of projection images using a color-vision-deficiency-safe palette. Right lungs labelled in yellow and left lungs labelled in cyan. Solid segmentation mask projections using maximum intensity projection (MIP) and color-coding of left and right lung are shown in the left column ("Colored MIP"). Standard deviation projections of the isosurface between foreground and background voxels of the binary segmentation masks using color-coding of left and right lung are shown in the right column ("Colored outline"). The underlying segmentations of rows 1-4 are identical to the one shown in Figure 3. The top row demonstrates a segmentation without significant errors according to the reference standard. The lower three rows show examples of segmentation errors: over-/under-segmentation of parts of the lung (row two), exclusion of lung pathology (consolidation) and off-target stitching (third row) and right-left-mislabeling of parts of the lung (bottom row). The presented yellow-cyan-visualisations were not assessed in the study.
